# Changes in Vitreoretinal Adhesion With Age and Region in Human and Sheep Eyes

**DOI:** 10.3389/fbioe.2018.00153

**Published:** 2018-10-24

**Authors:** Christopher J. Creveling, Jourdan Colter, Brittany Coats

**Affiliations:** Department of Mechanical Engineering, University of Utah, Salt Lake City, UT, United States

**Keywords:** peel, vitreous, retina, inner limiting membrane, posterior vitreous detachment, retinal detachment

## Abstract

While several studies have qualitatively investigated age- and region-dependent adhesion between the vitreous and retina, no studies have directly measured the vitreoretinal strength of adhesion. In this study, we developed a rotational peel device and associated methodology to measure the maximum and steady-state peel forces between the vitreous and the retina. Vitreoretinal adhesion in the equator and posterior pole were measured in human eyes from donors ranging 30 to 79 years of age, and in sheep eyes from premature, neonatal, young lamb, and young adult sheep. In human eyes, maximum peel force in the equator (7.24 ± 4.13 mN) was greater than in the posterior pole (4.08 ± 2.03 mN). This trend was especially evident for younger eyes from donors 30 to 39 years of age. After 60 years of age, there was a significant decrease in the maximum equatorial (4.69 ± 2.52 mN, *p* = 0.016) and posterior pole adhesion (2.95 ± 1.25 mN, *p* = 0.037). In immature sheep eyes, maximum adhesion was 7.60 ± 3.06 mN, and did not significantly differ between the equator and posterior pole until young adulthood. At this age, the maximum adhesion in the equator nearly doubled (16.67 ± 7.45 mN) that of the posterior pole, similar to the young adult human eyes. Light microscopy images suggest more disruption of the inner limiting membrane (ILM) in immature sheep eyes compared to adult sheep eyes. Interestingly, in human eyes, ILM disruption was significantly greater in the posterior pole (*p* < 0.05) and in people over 60 years of age (*p* < 0.02). These findings supplement the current discussion surrounding age-related posterior vitreous detachment, and the risk factors and physiological progressions associated with this condition. In addition, these data further our understanding of the biomechanical mechanisms of vitreoretinal adhesion, and can be used to develop age- appropriate computational models simulating retinal detachment, hemorrhaging, or retinal trauma.

## Introduction

Any disruption to the layers of the retina, particularly the separation of the photosensitive cells from the retinal pigment epithelium, can result in blindness or severe visual impairment. The most common cause of disruption is retinal detachment. Retinal detachment occurs in one of every 10,000 people (Mitry et al., 2010) and does not discriminate between children and adults (Rosner et al., 1987; Fivgas and Capone, 2001). Ocular trauma and age-related vitreous degradation are common causes of detachment, and the principal mechanism for each of these etiologies is retinal force mediated by adhesion to the vitreous.

Little is quantitatively known about adhesion at the vitreoretinal interface. Sebag (1991) manually peeled the posterior vitreous from the retina in 59 post-mortem eyes from donors with ages spanning 33 weeks gestation to 100 years old. They reported that peeling was “more difficult” in younger ages (20 years old or younger). Additionally, they report disruption of the Müller cells after peeling in 40% of the younger eyes, suggesting vitreoretinal adhesion in the younger group was stronger than adhesion between individual layers of the retina. This study provides evidence for changes in adhesion with age. It also correlates with theories that suggest vitreoretinal adhesion is a function of collagen density and structural integrity (Sebag, 1992; Fivgas and Capone, 2001; Gandorfer et al., 2001, 2002; Bishop et al., 2004; Ponsioen et al., 2008; Mitry et al., 2010). The amount of vitreal collagen present at birth does not change throughout life, so the relative density of collagen in the eye decreases as the eye grows (Balazs and Denlinger, 1982), and degrades with time (Bishop et al., 2004). If adhesion is dependent on collagen density and structure, the decrease and weakening of collagen with age would alter and diminish adhesion at the vitreoretinal interface.

Vitreoretinal adhesion has qualitatively been shown to vary with region. Gandorfer et al. (2001) injected plasmin into the vitreous of 24 porcine eyes and evaluated the vitreoretinal interface using scanning and transmission electron microscopy. Greater dosages and incubation times were required to eliminate and/or separate collagen fibrils on the inner limiting membrane (ILM) of the equator compared to the posterior pole. None of the dosages evaluated were able to completely separate the vitreous cortex from the ILM at the vitreous base. This suggests that vitreoretinal adhesion increases from the posterior pole to the equator to the vitreous base. The exact mechanism of adhesion is not well-established and may be different in the vitreous base compared to the equator or the posterior pole. It is possible the plasmin used in the Gandorfer study was more successful in detaching the vitreous from the retina in the equator and/or posterior pole because of regional adhesion mechanisms rather than adhesive strength.

No studies to date have directly measured adhesive forces at the vitreoretinal interface, but there are several studies that quantified retinal separation at the pigment epithelium (RPE). The earliest series of studies were by Zauberman and deGuillebon (Zauberman and Berman, 1969; DeGuillebon et al., 1971; DeGuillebon and Zauberman, 1972; Zauberman, 1972) in monkey, cat, and rabbit eyes. Both groups excised rectangular specimens from the eye containing retina, choroid, and sclera. The specimens were laid flat in a saline-filled petri dish. A metallic rod was attached to the ILM of the retina and pulled either manually (Zauberman and Berman, 1969), or with a computer-controlled linear actuator (Zauberman, 1972). The adhesive forces between the RPE and choroid were higher in the equator compared to the posterior region (Zauberman and Berman, 1969), and this regional dependence was reported to be greater in younger rabbits (1–2 months old) compared to adult rabbits (DeGuillebon et al., 1971). Additional experiments report significantly increased RPE adhesion with peel rate (DeGuillebon et al., 1972) and significantly decreased adhesion with post-mortem time (Zauberman and DeGuillebon, 1972). A subsequent study by Endo et al. (1988), however, reported that refrigeration of enucleated eyes delayed deterioration up to at least 18 h.

The limitation of these peel studies is the potential disruption of the interface between the RPE and choroid prior to testing. Sandwich specimens were physically removed from the spherical eye and laid flat for testing. The excision likely damages structures at the cut interface, and straightening the sample likely causes shear forces between the layers which may compromise the interface. To overcome these limitations, Kita et al. (1990) used a bleb technique to estimate adhesion at the RPE. Briefly, a balanced salt solution was slowly injected into the subretinal space to generate retinal separation and a fluid-filled bleb. The choroidal retinal adhesive force was estimated from measured pressure differences between the vitreous and the fluid-filled subretinal space. The accuracy of these calculations is based on the assumption that the fluid-filled bleb is spherical. Previous bleb studies have reported that subretinal injections typically result in blebs with flattened rather than spherical tops (Marmor et al., 1980). The error this may cause in adhesion force estimations is unknown.

The objective of our study was to quantify vitreoretinal adhesive forces in sheep and human eyes, and evaluate how measurements change with age and region of the eye. To achieve this objective, an innovative testing device was created to overcome many of the limitations of the previous retinal adhesion methods. This device allows the retina to be peeled from the vitreous without altering the curvature of the specimens, or requiring dissection of the retina. Using this technique, quantitative vitreoretinal adhesive forces can be directly measured. These forces will be necessary to understand the biomechanics of vitreoretinal adhesion and create numerical tools for predicting retinal detachment.

## Methods

### Materials

The adhesive strength between the vitreous and retina was measured in sheep (*n* = 43) and human (*n* = 17) eyes. Sheep eyes were used to investigate differences in adhesion between immature and mature eyes. Sheep eyes were selected because they have a well-defined retinal structure and holangiotic vasculature, and their vitreous composition is similar to human eyes (Balazs and Denlinger, 1982; Sebag, 1993; Ponsioen et al., 2010). Of the sheep eyes, four age groups were compared: adult (*n* = 15, 4–6 years old), young lamb (*n* = 10, 18 weeks old), neonatal (*n* = 5, 1–5 days old), and premature (*n* = 13, 128–136 days gestational age). There are no known human age equivalents for sheep based on ocular anatomy. The young adult sheep used in this study have a human age equivalence of 28–36 years old based on reproductive maturity and life span (Lévy et al., 2017). The immature groups (young lamb, neonatal, and premature) do not have well-established age equivalencies. Sheep brain development peaks *in utero* at ~85 days gestation (Dobbing, 1974). Based on the brain development patterns, our premature and neonatal groups are estimated to have human equivalent ages of 6–12 and 14–20 months old, respectively. The young lamb group is estimated to have a human equivalent age of a 5–7 years of age. This lamb age equivalency was estimated based on extrapolation of the early brain maturation data and reproductive maturity timelines.

Sheep eyes were removed immediately upon sacrifice and refrigerated *en bloc* in phosphate-buffered saline (PBS) until testing. Adult human eyes (30–80 years old) were purchased from the Utah Lions Eye Bank in PBS and refrigerated until time of testing. Left and right eyes for all sheep and human subjects were collected and tested within 24 h of death. All studies were reviewed by the University of Utah IRB and IACUC compliance boards and determined to be exempt from regulation.

**Figure 1:**
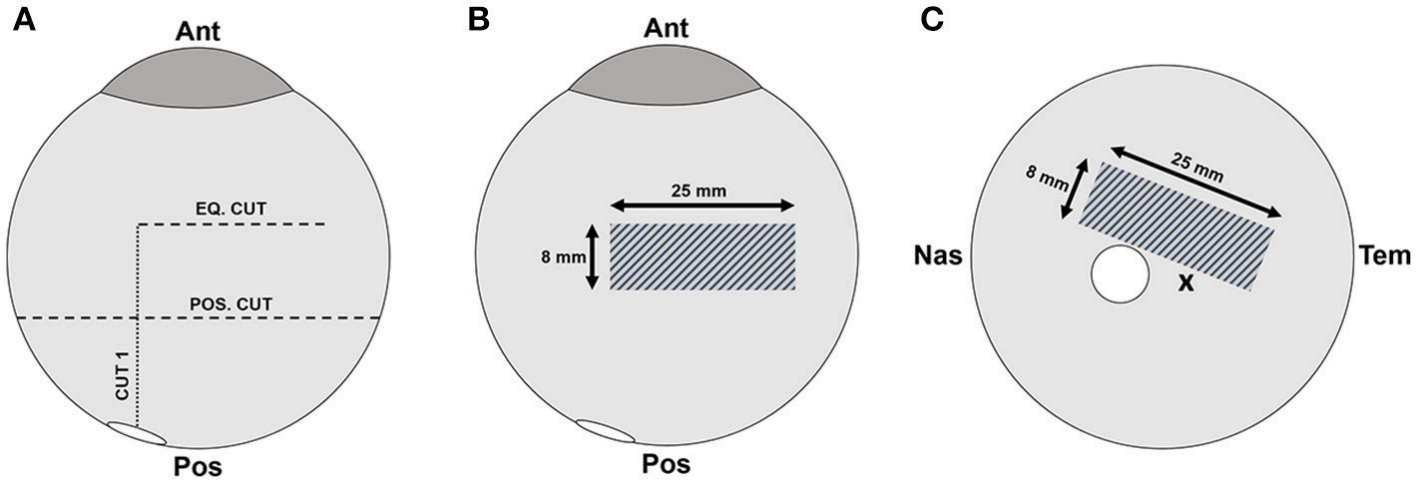
**(A)** Scleral dissection methods used to create a rectangular retinal window (gray crosshatch) in the **(B)** equatorial and **(C)** posterior regions. The location of the optic nerve head is shown as a white circle, and the fovea marked with an “**x**”.

Testing was performed in the equatorial and posterior pole regions for each eye. The order in which samples were tested (right/left, equator/posterior pole) was randomly selected. Extraocular tissue and the optic nerve were removed from the globe prior to all dissections. For equatorial peels, a cut through the sclera, leaving the choroid, retina, and vitreous intact, was made anteriorly from the small opening at the optic nerve head to the equatorial region, ending ~15 mm posterior to the cornea (Cut 1 in Figure 1A). A second cut was made perpendicular to the initial cut, and along the equatorial region of the eye, ~25 mm in length (Eq. Cut in Figure 1A). Third and fourth cuts were made to create an 8 × 25 mm rectangular window of choroid (Figure 1B). Forceps were used to carefully pull away the choroid and expose the underlying retinal pigment epithelium.

For posterior peels, an initial cut was made similar to the equatorial peels (Cut 1 in Figure 1A). A circumferential cut was made using forceps and dissection scissors around the globe along the posterior cut line (Pos. Cut in Figure 1A), and the posterior sclera was removed entirely from the globe. This provided better visualization and access to the retina in the posterior pole. The choroid was removed using forceps and dissection scissors. The posterior retinal testing region was always oriented at an angle to avoid the fovea (Figure 1C).

### Peel test setup and protocol

**Figure 2:**
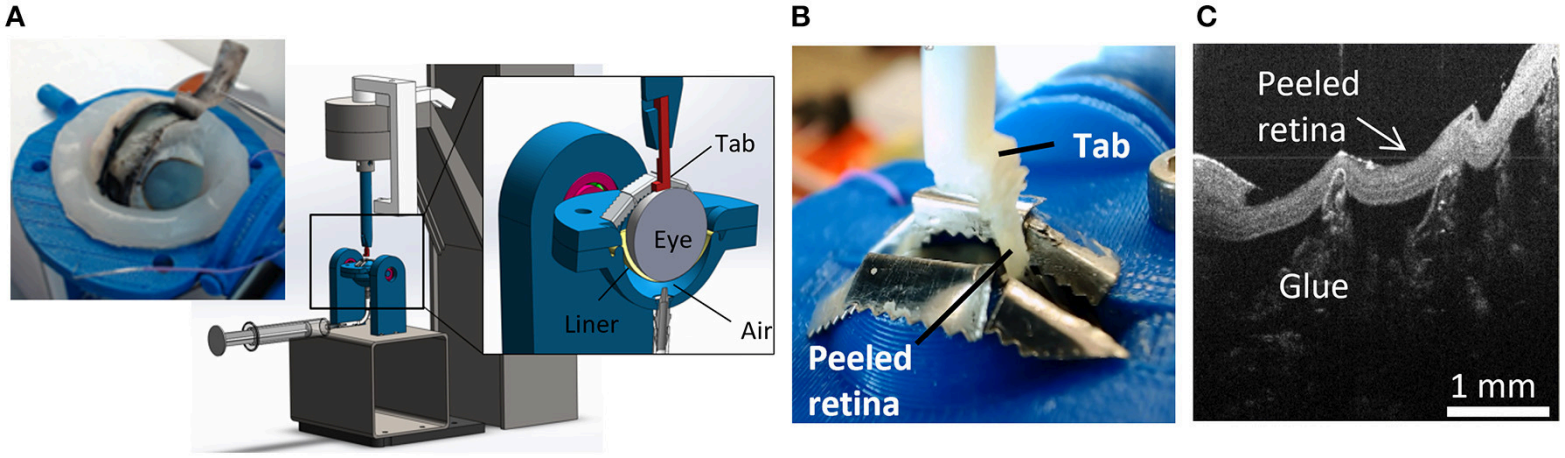
For each peel test, **(A)** sclera was dissected away from the retina prior to testing, and the eye placed in the fixture. The eye was raised toward the testing window by inflating a polymer liner with air. **(B)** An L-shaped tab was glued to the retina and raised to peel the retina from the vitreous while measuring force. **(C)** Optical coherence tomography image of the peeled retina indicating separation was at the vitreoretinal interface and glue penetration was minimal.

A peel test system was created to test vitreoretinal adhesion with minimal dissection and disruption to the vitreoretinal interface. This was achieved by keeping the retina and vitreous in their natural configuration, and rotating the eye as the retina was peeled away from the vitreous. The prepared eye with a window of exposed retina was placed onto a flexible membrane (Dragon Skin, Smooth-On, Macungie, PA) molded to cup the eye (Figure 2A). The eye and membrane were loaded into a custom 3D-printed holder with a rectangular opening that lined up with the exposed retina. Air was pumped into the cavity beneath the flexible membrane to enforce a spherical shape at the vitreoretinal interface. Two different sizes of membranes and eye holders were designed. The large size (diameter = 30 mm) was used to hold adult and young lamb sheep eyes. The small size (diameter = 20 mm) was used for neonatal and preterm sheep and human eyes.

A plastic L-shaped tab was connected to an Instron Universal Testing Instrument (Model No. 5943, Instron, Norwood, MA) where load measurements were made with a 5 N uniaxial load cell (Model No. 2530-5N, Instron, Norwood, MA). A thin layer of cyanoacrylate adhesive was applied to the bottom surface of the tab (5.0 × 4.5 mm) and lowered until it was in contact with the retina (Figure 2B). The retina was scored on both sides of the tab as well as one end of the peel region to ensure a clean rectangular peel shape throughout the duration of the test. The tab was raised at a quasistatic rate of 0.02 mm/s in accordance with other previous peel test literature (DeGuillebon and Zauberman, 1972; Zauberman and DeGuillebon, 1972; ASTM, 2016). The eye holder rotated simultaneously using a pulley system connected to the Instron crosshead. This rotation was critical for maintaining a constant perpendicular angle of peel during testing. Tests lasted anywhere from 5 to 12 min depending on the length of the steady-state peel. A Logitech web cam (Logitech C920 HD Pro, Logitech, Newark, CA) recorded video of each peel test. Optical coherence tomography (Envisu R2200, Leica Microsystems, Wetzlar, Germany) imaging was used to verify the glue between the tab and the retina did not penetrate deep into the retina and affect adhesion measurements (Figure 2C).

Following rotational peel tests, 2 mm square sections of peeled retina not directly attached to the tab were removed to characterize damage to the vitreoretinal interface. Specimens were placed in a 1% buffered formaldehyde and 1.25% glutaraldehyde solution for 24 h. For processing, samples were placed in a 0.1 M sodium cacodylate buffer rinse twice for 5 min while being agitated. Samples were then placed in a 1:1 mixture of diluted osmium tetroxide *OsO*_4_ (4% in *dH*_2_*O*) and 0.2 M sodium cacodylate buffer and agitated for 1 h. The samples were rinsed with DI water to remove excess osmium, and then placed in a 4% uranyl acetate solution, and agitated for 1 h. The samples were then dehydrated in increasing percentages of ethanol alcohol (50, 70, 95, and 100%) and then placed in acetone. Processed specimens were infiltrated using unpolymerized resin plastic and cured at an elevated temperature in an oven overnight. Specimens were cut to 0.5 μm thick slices, stained with toluidine blue, and imaged on an upright microscope (Olympus CX41, Olympus, Center Valley, PA).

### Peel test validation

**Figure 3:**
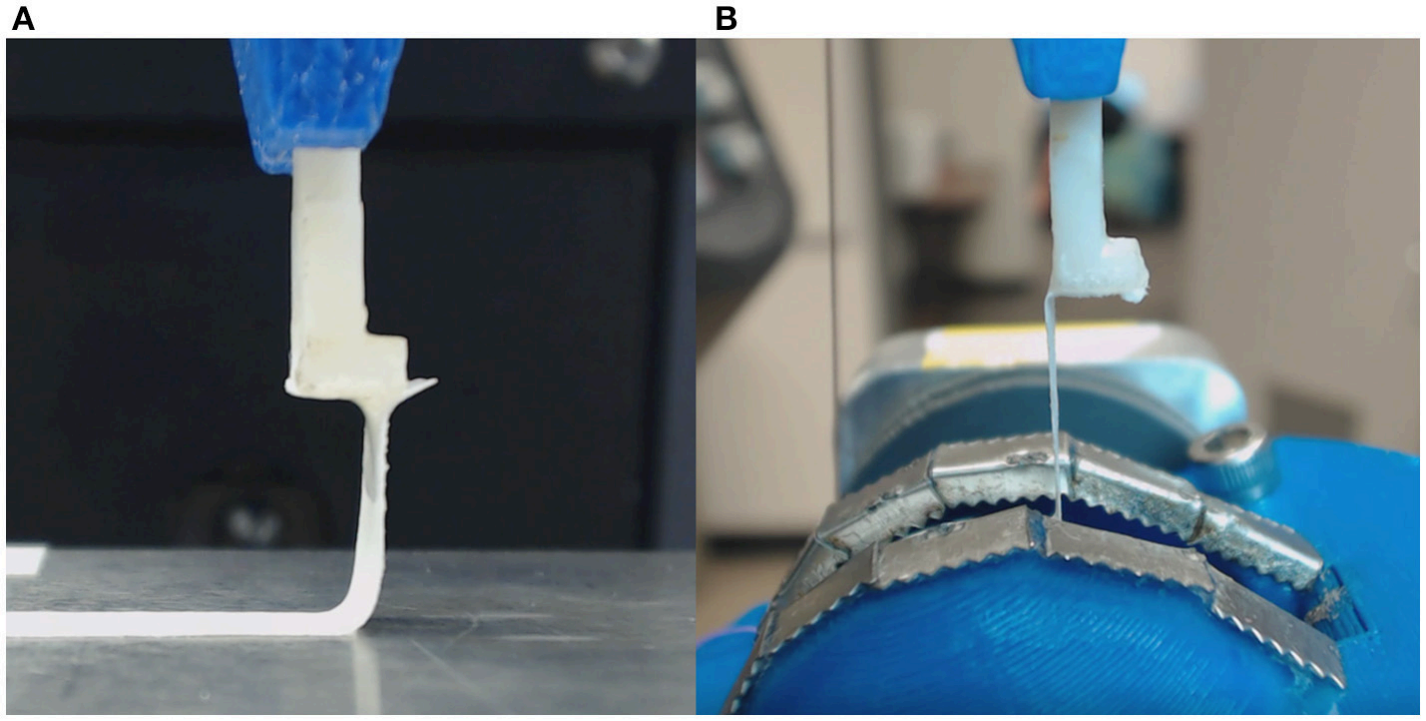
**(A)** Tape linear peel tests were performed and compared to **(B)** tape rotational peel tests to ensure validity of the custom rotational peel device.

The novel rotational peel system was validated by measuring adhesion between metal and tape (Daigger, Vernon Hills, IL) and comparing measurements in the rotational system to measurements from linear peel testing using ASTM standard D6862-11 (Figure 3A; ASTM, 2016). For the rotational test, tape was adhered to a aluminum ball bearing and placed inside the eye holder (Figure 3B). The tape was glued to the tab similar to the retinal experiments and followed the protocol described in section Peel Test Setup and Protocol. The linear peel test setup was identical to the rotational test, however it differed by adhering the tape to a flat aluminum surface (Figure 3A). After gluing the tab to the tape, the aluminum plate was horizontally actuated as the tab moved upward to maintain a 90° peel angle. The adhesive force over time for the rotational (*n* = 6) and linear (*n* = 3) peel tests were compared.

### Data analysis

In any peel test, a maximum force is required to initiate a peel, and a steady-state peel force maintains the peel. In this study, these attributes were extracted through careful examination of videos of each peel test in conjunction with the force-time data.

The maximum peel force was defined as the maximum force measured prior to separation between the retina and the vitreous. The period of steady-state peel was defined as the time period of relatively constant force after the vitreous separated from the retina, leaving vitreous in contact at the base of the peel. A video showing a typical peel test is provided as Supplemental Data. The steady-state peel force was calculated as the average force across the steady-state peel period. Retinal samples that detached from the L-shaped tab or tore before steady-state peeling were excluded from analysis.

A two-way ANOVA with repeated measures was performed to evaluate the effect of region and age on the maximum and steady-state peel force in the sheep eyes and human eyes. Repeated measures were used because regional data was collected from the same eyes. To perform this test in humans, donor ages were binned into the 4, 5, 6, 7, or 8th decade of life. Tukey-Kramer *post-hoc* tests were selected to evaluate pairwise comparisons within each effect. A linear regression was also performed in the human eyes to see if the maximum and steady-state peel force significantly decreased with age. The linear regression analysis was executed separately for each region (equator, posterior pole).

To identify significant differences in mechanisms of failure, the light microscopy images of the peeled retina were rated according to the following criteria: 0–ILM cleanly separated with no disruption or evidence of traction; 1–ILM cleanly separated, but ILM is undulated or there is evidence of traction on the ILM; 2–ILM cleanly separated, with the exception of 1–2 small localized disruptions (typically around vessels); 3–ILM torn and disrupted. A chi-square test was performed on these scores to identify significant differences between region and age. A logistic regression was also used to determine if the maximum and steady-state peel forces were predictive of failure type (i.e., 0, 1, 2, or 3). For all statistical tests, *p*-values < 0.05 were considered significant.

## Results

**Figure 4:**
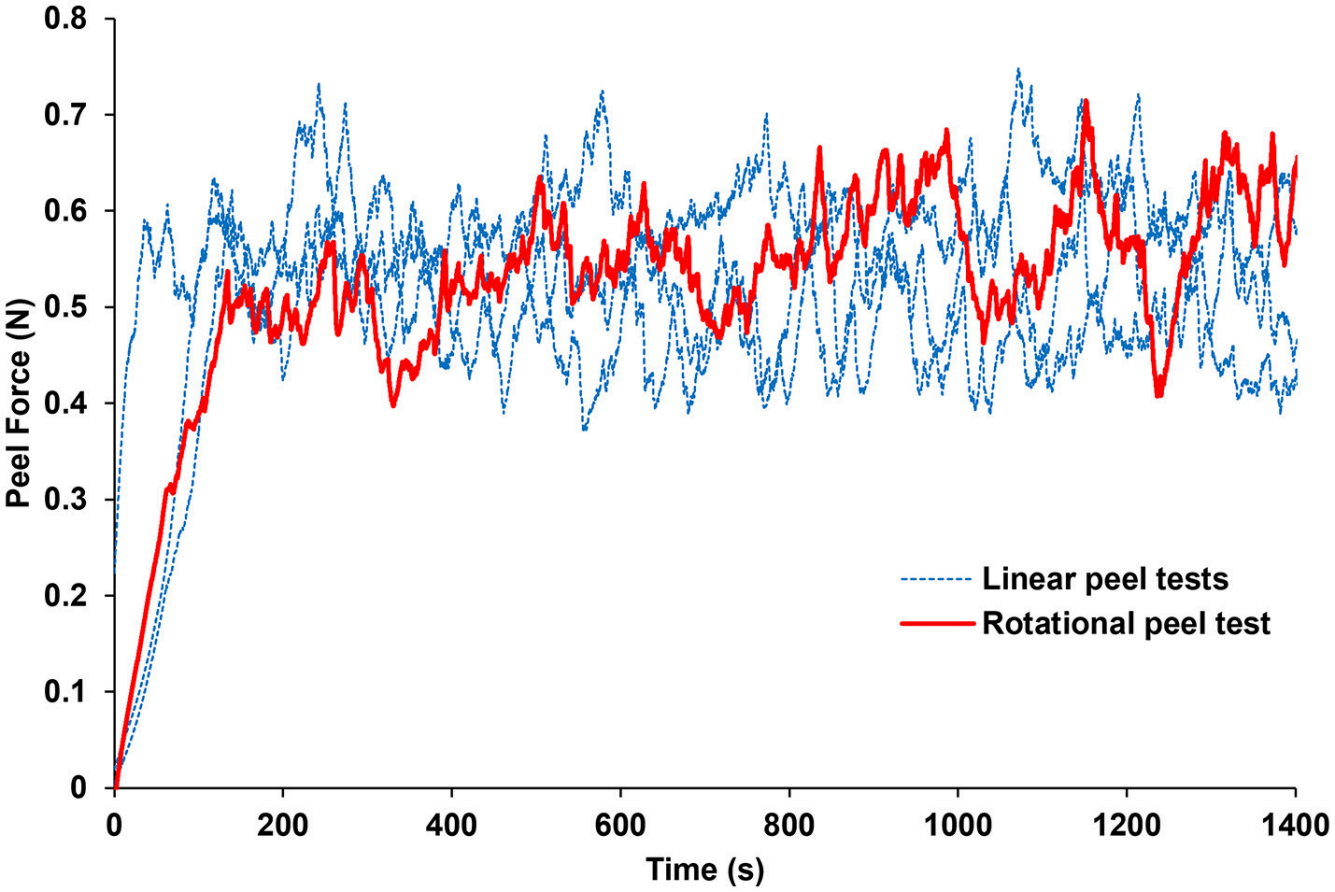
Average peel force measurements from the rotational tests were within 1.3% of the average peel forces measured from linear peel tests using ASTM standard D6862-11. Only one rotational peel test is plotted for clarity.

**Figure 5:**
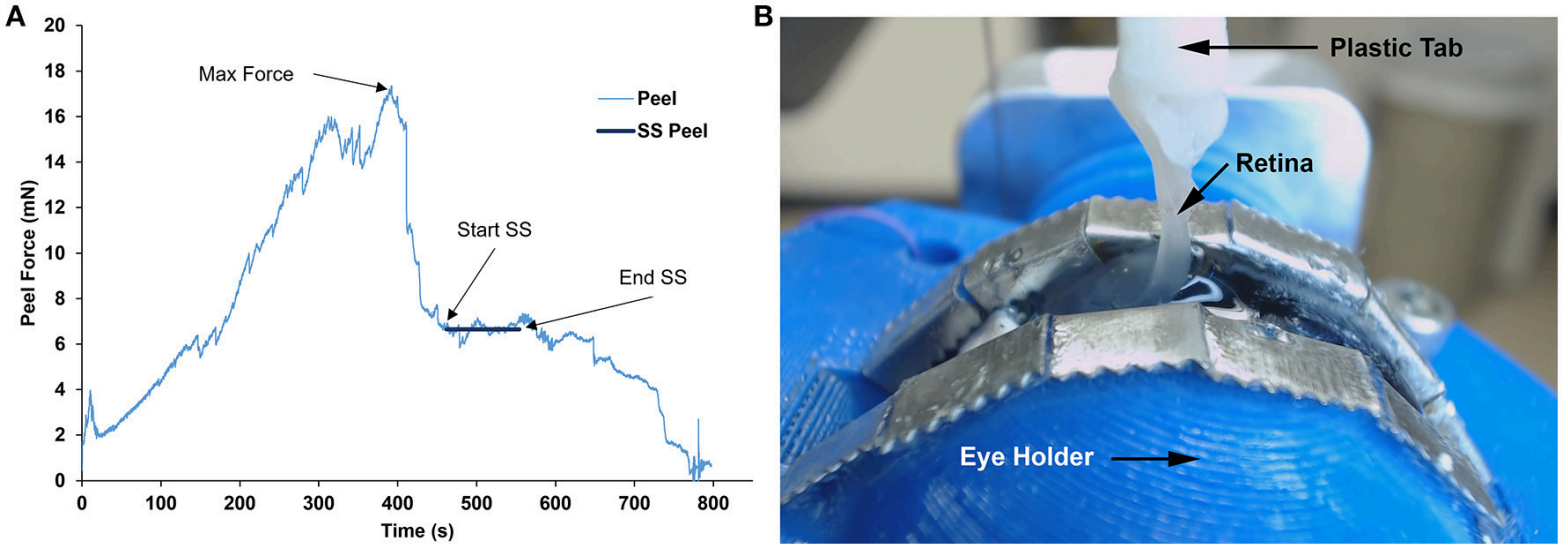
**(A)** Representative peel test showing peel force over time for a sheep specimen. Steady-state (SS) peel region started after maximum peel force was reached and ended before the retina began to fail. Steady-state peel force was calculated by averaging forces during the steady-state peel period. **(B)** Still image taken from the webcam video of a sheep retina peeling away from the vitreous at 90° during the steady-state peel region.

The forces measured from the linear and rotational tape peel tests did not exhibit a definitive peak force (Figure 4), so steady-state peel force was calculated as the average of the data after the initial ramp up period. The resulting linear and rotational steady-state peel forces were 0.534 ± 0.062 and 0.527 ± 0.075 N, respectively, validating the device and methodology. All human and sheep peel tests exhibited a distinct maximum peel force (Figure 5A) followed by a steady-state peel region (Figure 5B). Decreases in force after the steady-state period were due to defects in the retina causing it to prematurely tear, retinal thinning or stretching during particularly long peel tests, or manual cutting by the test observer to end the test.

### Effect of age and region in sheep eyes

**Figure 6:**
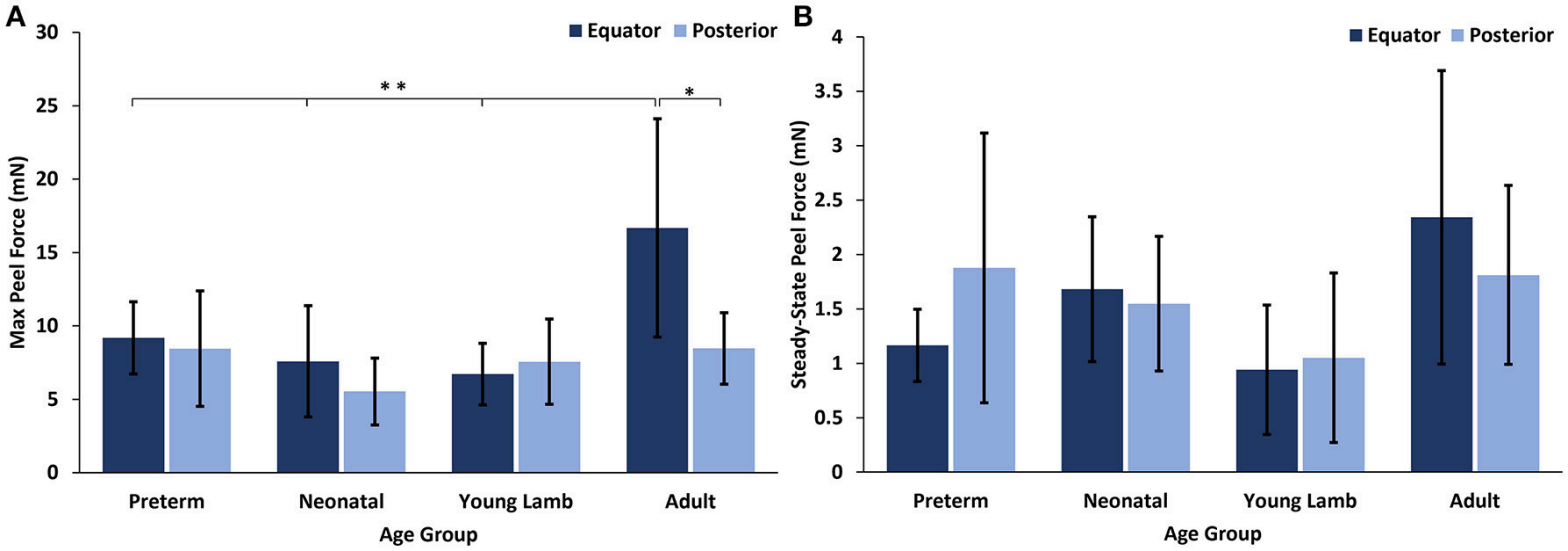
**(A)** Maximum and **(B)** steady-state peel forces for each sheep age group and region. Error bars indicate standard deviation from the mean (*p = 0.0016 and **p < 0.0001).

No significant differences with region were seen for either the max or steady-state peel force except for the adults which had significantly higher max peel force in the equatorial region (16.67 ± 8.46 mN) compared to the posterior region (8.46 ± 2.43 mN, *p* = 0.0016, Figure 6). Maximum peel force in the equator was also significantly higher in the adult compared to the younger ages (7.96 ± 2.81 mN, *p* < 0.0001). In most premature eyes, the steady-state adhesion in the posterior pole (1.88 ± 1.24 mN) was greater than the equator (1.12 ± 0.33 mN), but this finding was not statistically significant.

### Effect of age and region in human eyes

**Figure 7:**
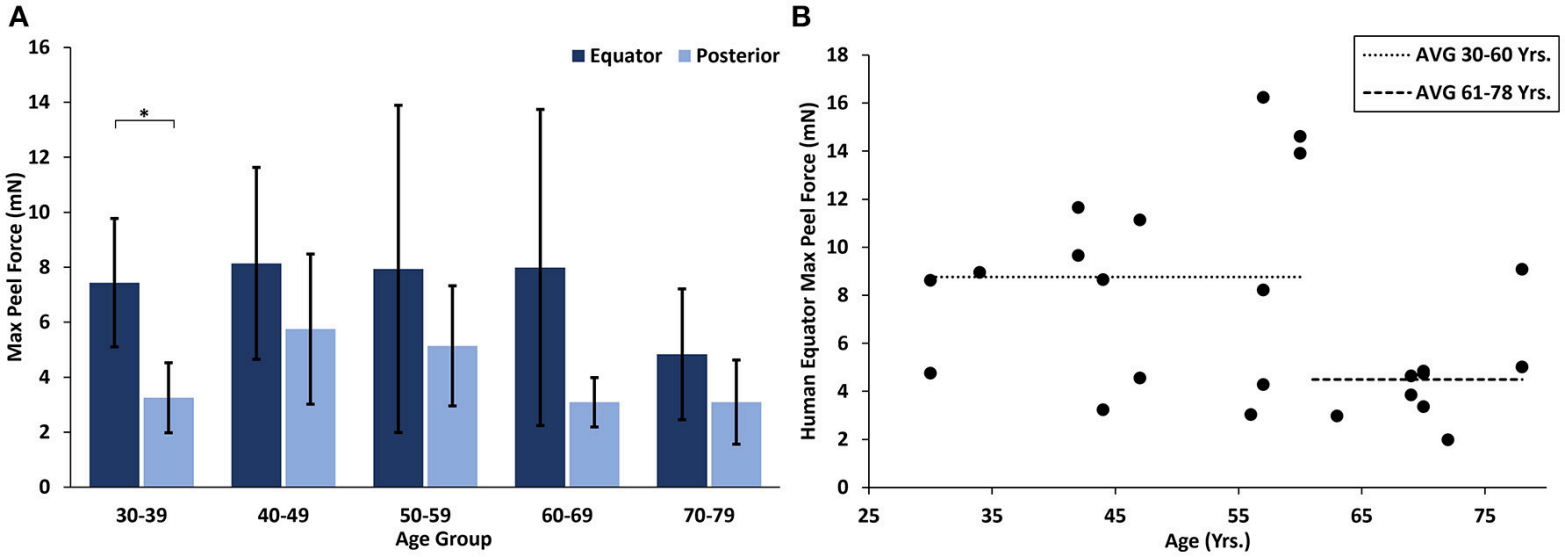
**(A)** Maximum peel force for human specimens by age group and region. Error bars indicate standard deviation from the mean (*p < 0.03). **(B)** Equatorial maximum peel force was significantly reduced after 60 years of age (*p* < 0.01). Bars indicate mean data prior to and including 60 years of age, and after 60 years of age.

**Figure 8:**
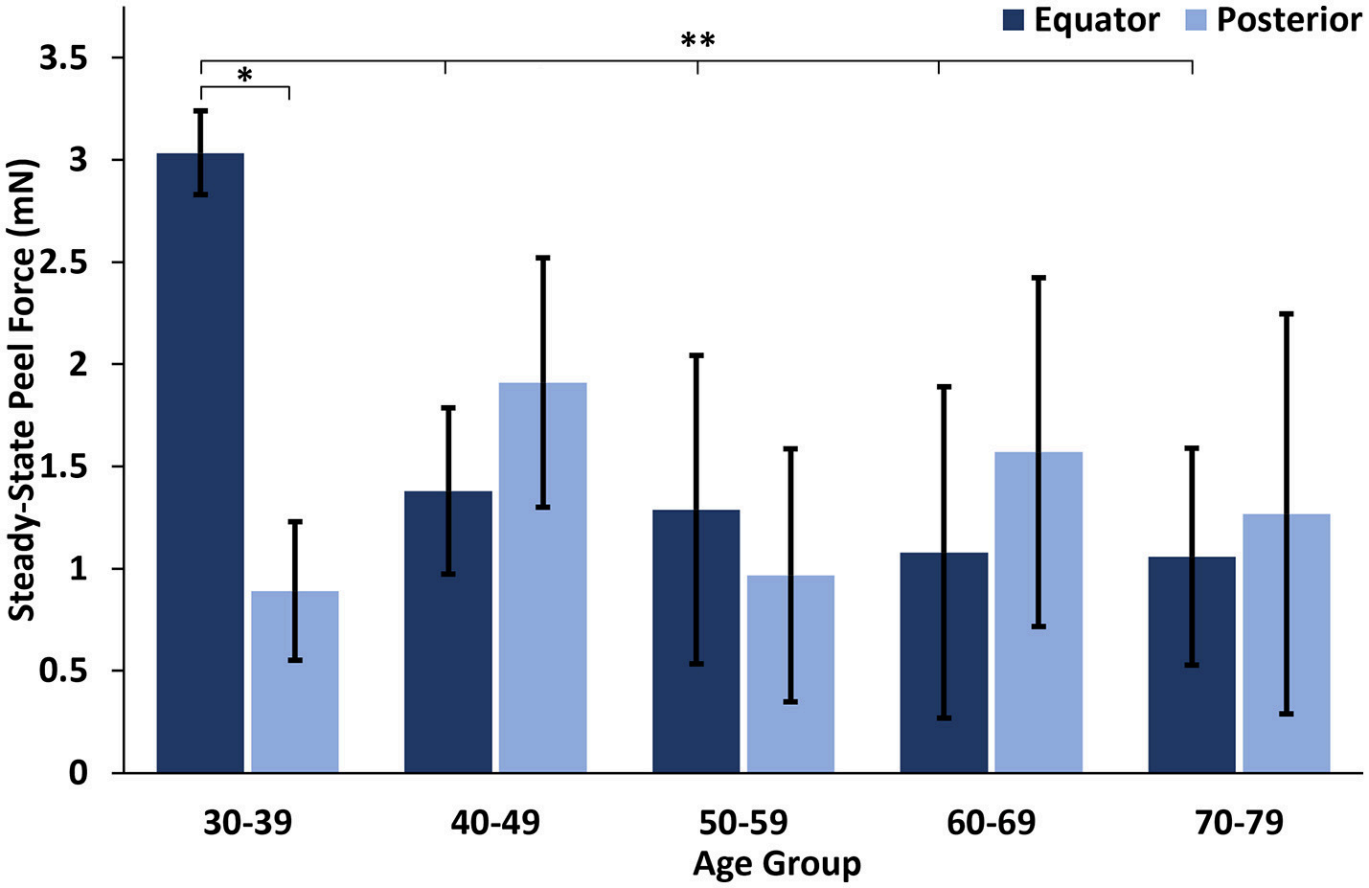
Steady-state peel force in the equator of the youngest human eyes was significantly greater than the posterior pole, and significantly greater than the equatorial steady-state adhesion of all other ages. Error bars indicate standard deviation from the mean. *p < 0.002 and **p < 0.015.

At all ages, the maximum peel forces in the equator (7.16 ± 4.08 mN) were greater than those in the posterior pole (4.08 ± 2.03 mN, Figure 7A), but this regional difference was only significant in the youngest adult ages (*p* < 0.03). With age groups defined by decade of life, there was no significant difference in maximum adhesion in the equator or posterior pole with age. This finding was confirmed by the linear regression with age. However, when maximum peel force was plotted against age, there was a noticeable decrease in equatorial vitreoretinal adhesion after 60 years of age from 8.76 ± 4.22 to 4.50 ± 2.00 mN (Figure 7B). This decrease was found to be significant when evaluated with a Student's *t*-test (*p* < 0.01). Steady-state peel force was significantly affected by age (*p* < 0.006), region (*p* < 0.0001), and their interaction (*p* < 0.001). Specifically, vitreoretinal adhesion in the equator of the youngest adults (7.44 ± 2.34 mN, 30–39 years old) was significantly greater than the posterior pole in that age group (3.25 ± 1.27 mN, *p* < 0.002) and significantly greater than the equatorial adhesion in the other age groups (*p* < 0.015, Figure 8).

### Locations of failure

**Figure 9:**
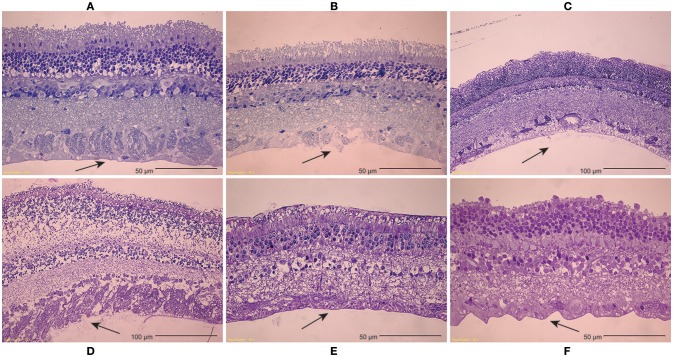
**(A–C)** Light microscopy images of peeled sheep retinas indicate **(A)** failure primarily occurred at the ILM. **(B)** In some images, focal ILM disruption was noted, **(C)** particularly in the presence of blood vessels. There were no regional differences in failure location, but immature sheep eyes tended to have more ILM disruption than young adult sheep eyes. **(D–F)** For human eyes, **(D)** peeling in the posterior pole typically disrupted the ILM, **(E)** while peeling in the equator had a clean separation between the vitreous and retina. **(F)** This clean separation was sometimes accompanied by traction-like undulations on the retinal surface.

Inspection of light microscopy images in the sheep revealed generally clean separation at the vitreoretinal interface (Figure 9A). Occasional disruption or failure of the ILM was observed (Figure 9B), especially in the presence of blood vessels (Figure 9C) and in immature eyes (i.e., premature, neonatal, and young lamb). Retinal stretching without ILM disruption was often seen in the nerve fiber layer, ganglion cell layer, or the outer plexiform layer. These observations were not different between the equator and posterior pole. In the human eyes, large disruption of the ILM occurred in the posterior pole (Figure 9D), and was significantly different than the failure location in the equator (*p* < 0.05) which exhibited a clean separation (Figure 9E), oftentimes with indication of tractional pulling (Figure 9F). ILM disruption in the posterior pole was significantly greater in eyes from donors > 60 years of age (*p* < 0.02). No significant differences in failure location were found with gender, and ILM disruption was not predicted by the maximum or steady-state peel forces.

## Discussion

In this study, we developed a novel rotational peel test system to measure the strength of adhesion at the vitreoretinal interface. We used the device to quantify age- and region-dependent vitreoretinal adhesion in human and sheep eyes. These data are critical to improving biomechanical understanding of the vitreoretinal interface. They can be implemented into computational models to investigate posterior vitreous detachment, or to simulate traumatic ocular injury. Further, the peel test system and associated data can be used to evaluate the effectiveness of chemical compounds to assist in surgical separation of the retina and vitreous.

In both sheep and human eyes, there was an interesting distinction between the maximum force of vitreoretinal adhesion in the equator and posterior pole that was highly dependent on the maturity of the animal. In premature, neonatal, and young lamb eyes, there were no regional differences. By the time the sheep developed into young adults (~28–36 years human age equivalent), the equatorial vitreoretinal adhesion significantly increased while adhesion in the posterior pole remained unchanged from the immature sheep eyes. This may be due to posterior extension of the vitreous base and ILM thickening with age. Wang et al. investigated the anteroposterior length of the vitreous base in 58 pairs of human eyes from subjects spanning ages of 8–96 years old (Wang et al., 2003). They report a continual posterior extension of the length of the vitreous base until ~80 years old, at which time the length decreases. A similar study has not been performed in sheep, but a posterior extension of the vitreous base with age would explain the increased equatorial adhesion in the adult sheep eyes compared to immature sheep eyes.

Similar to the sheep eyes, the maximum peel force in the equator of the human eyes was greater than in the posterior pole. This was only significant in the youngest age group (30–39 years old), but all binned age groups younger than 70 years of age had higher average adhesion in the equator than in the posterior pole. One interesting finding from our dataset was a significant decrease in equatorial maximum peel force in eyes from human subjects > 60 years of age (Figure 7B). This suggests the existence of age-related changes that reduce equatorial adhesion, and overshadow any increased adhesion due to vitreous base extension. The distinct drop-off in equatorial max peel force around 60 years of age corresponds with the timeline of vitreous liquefaction (Foos, 1972; Balazs and Denlinger, 1982; Sebag, 1987, 1989; Uchino et al., 2001) and may be protective to the retina by facilitating posterior vitreous detachment (PVD) before retinal tearing or retinal detachment occurs. In PVD, vitreoretinal separation is typically thought to begin near the macula and progress anteriorly (Kakehashi et al., 1997, 2014). As liquefaction occurs, increased traction is placed on the retina at locations of strong adhesion. The decreased equatorial adhesion after 60 years of age, a time when PVD is most prevalent, reduces the risk of retinal tearing or detachment in the equator. Further to this point, the steady-state peel force in our study was similar in both the posterior pole and the equator for all ages except the youngest age group (30–39 years old). This suggests that for most adults, PVD can gradually develop anteriorly into the equatorial region with minimal resistance and risk for retinal tearing. However, in young ages, there will be an increased risk for retinal tearing or detachment. This may explain why severe myopes, who have an increased disposition to liquefaction at young ages, have an increased risk for retinal detachment (Akiba, 1993).

Our study is the first quantitative measurement of vitreoretinal adhesion, but other groups have qualitatively evaluated adhesion at the vitreoretinal interface (Sebag, 1992; Marmor et al., 1994; Gandorfer et al., 2001). Their observations of changes in adhesion with age or region correlate with our findings. Sebag (1992) microscopically examined vitreoretinal adhesion in the posterior pole in human eyes from donors aged from 33 weeks of gestation to 94 years. He reported disruption to Müllers cells during peeling in 6 of the 15 eyes from donors < 20 years old, suggesting young adults and children have stronger posterior pole adhesion than older adults. In sheep eyes, we found no significant difference in posterior pole adhesion between immature and adult sheep. The young adult sheep in our study correspond to 26–38 years old for humans. We visually observed minimal to no vitreous liquefaction during dissection of the eyes, similar to the visually observed levels of liquefaction in our youngest group of human eyes (30–39 years old). If we had used older adult sheep, we may have seen distinctions in the posterior pole adhesion similar to Sebag.

Microscopic examination of peeled sheep retinas suggested generally clean separation at the ILM, regardless of age or region. Occasionally, the ILM was torn in immature eyes, regardless of region. In adult sheep, the ILM was never torn, but the nerve fiber layer was often stretched and occasionally disrupted locally around blood vessels. There was a single premature specimen with failure occurring in the ganglion cell layer, which is similar to Sebag. We obtained light microscopy specimens from 37% of our sheep peel tests. A more extensive microscopic analysis might correlate more closely with observations by Sebag.

In human adult peeled retinas, there was significant distinction between failure in the equator and posterior pole. The peeled retina in the equator was generally smooth with some undulation or traction-like peaks on the surface of the ILM, while many of the posterior peeled retinas exhibited complete disruption of the ILM. This was surprising given that adhesion in the posterior pole is significantly lower than the equator. We hypothesize that numerous collagen penetrations in the posterior pole create large disruptions of the ILM when pulled, and that there is less collagen penetration in the equator where adhesion is thought to be dominated by adhesive proteins acting as an extracellular glue. Failure of this “glue” would result in cleaner separation. The traction-like characteristics may be caused by sparse penetration of collagen fibrils. Interestingly, ILM disruption in the posterior pole was significantly greater in eyes from donors older than 60 years of age. Because the adhesive strength is weak at this age, we hypothesize that the disruption of the ILM is an indication of decreased retinal structural integrity. Regardless, these data show adhesive strength cannot be inferred solely from locations of damage. Mechanism of adhesion and changes in retinal structural integrity with age likely contribute to patterns of failure.

Gandorfer et al. evaluated the effect of plasmin in different regions of porcine eyes (Gandorfer et al., 2001). After the same dosage and incubation time (2 U/0.1 mL plasmin, 60 min), they report a bare ILM in the posterior pole, sparse collagen fibrils in the equator, and a dense network of collagen fibrils at the vitreous base. This may suggest that adhesion is greater anteriorly to posteriorly, but different mechanisms of adhesion may occur in different regions of the eye, and may be affected by plasmin differently. Plasmin is known to digest fibrin and laminin, which are more prominent in the posterior pole compared to the equator (Mitry et al., 2010). There is some indirect digestion of collagen, but the extent is unknown. Future clarification is still needed to elucidate the role of glycoproteins in adhesion of the vitreoretinal interface at the vitreous base, equator, and posterior pole.

Several studies have quantitatively measured adhesion between the neurosensory layer and retina pigment epithelium (RPE) (Zauberman and Berman, 1969; Lincoff et al., 1970; DeGuillebon et al., 1971, 1972; DeGuillebon and Zauberman, 1972; Zauberman, 1972; Zauberman and DeGuillebon, 1972; Zauberman et al., 1972; Owczarek et al., 1975; Kain, 1984). Similar to our results, these studies report significantly greater adhesion in the equator compared to the posterior pole. Zauberman and Berman (1969) reported 1.18–2.45 mN in the equator compared to 0.59–0.88 mN in the posterior pole in cats. DeGuillebon et al. (1971) reported 0.77–1.41 mN in the equator compared to 0.86–1.206 mN in the posterior pole in rabbits. One exception to this is a study by DeGuillebon et al. (1972) that found increasing peel rates increased RPE adhesion in the posterior pole compared to the equator. The rates used in the present study are quasistatic, and suitable for PVD investigations. Additional vitreoretinal adhesion studies at higher rates will need to be performed to understand region and age-related differences associated with ocular trauma.

Retinal detachment or tearing can occur due to vitreoretinal traction, so adhesion at the vitreoretinal interface is likely greater than adhesion at the RPE in healthy young adults. No studies have measured RPE adhesion in sheep or in humans, making comparison of our measurements with the literature challenging. Kita and Marmor (1992) used subretinal injections to calculate posterior RPE adhesion in young adult primates (3.8–7.9 kg). They report average adhesive forces of 140 ± 3 dynes/cm using a subretinal bleb technique. Without a reported bleb circumference, it is impossible to convert their measurements to mN and compare to our study. However, they state primate adhesive forces were 140% greater than that of rabbits, and an earlier study by the same group (Kita et al., 1990) reported 1.979 ± 0.22 mN (converted using reported bleb circumferences). This results in an approximate primate RPE adhesion of 2.771 ± 0.31 mN, and is 1.7 times lower than the average adhesion human eyes from donors 30–60 years of age in our study. Of note, the RPE adhesion measured in primates was only 10% lower than the maximum posterior peel force in our oldest age group (70–79 years old). None of the eyes we tested had PVD, so this oldest age group likely had greater vitreoretinal adhesion than those at risk for PVD.

In standard peel tests, a thin membrane is typically peeled from a solid surface. In these studies, we peeled a thin membrane (retina) from a gel (vitreous). This resulted in deformation of both materials prior to and during peeling. The maximum peel forces measured in this study were defined as the maximum force before clear separation of the vitreous from the retina. With this definition, the maximum peel force may incorporate some retinal stretching or separation from the scored retinal edges in addition to the peeling force. During steady-state peeling, the retina also experienced some deformation, however, careful examination of video in conjunction with the peel force data provided confidence that peeling, and not retinal deformation, was the primary contributor to the steady-state measurements. A computational simulation of the peel tests is planned for a future study to separate tissue adhesion and vitreous deformation in the maximum peel force measurements.

The data collected in this study was on the low end of the load cell limit. Because of this, we had the load cell carefully calibrated at its reported lower limit of 9 mN. The uncertainty measurement at this limit was 0.002 gf, or 0.0196 mN, and decreased with decreasing load. The maximum peel force data in our study ranged from 4 to 15 mN, which is near the lower limit of the load cell. The steady state peel forces were lower than the maximum peel forces (1–3 mN), but were still on the same order of magnitude as the calibrated limit, and still two orders of magnitude larger than the uncertainty measurements. It is possible the load cell limits contributed to the variability in the steady-state measurements, but we have strong confidence in the conclusions and trends of the study.

Vitreoretinal adhesion to large blood vessels is thought to be greater than adhesion in regions without blood vessels. Our observations of ILM tearing surrounding blood vessels support this notion. Further, we observed steady-state peel forces drop after passing a blood vessel. For this study, we extracted steady-state values from regions without the blood vessels in order to maintain a consistent comparison across all ages and regions. A comprehensive and focused assessment of the effect of blood vessels on adhesion will be performed in a future study.

## Conclusion

We developed a novel device to quantify vitreoretinal adhesion in the equator and posterior pole of human and sheep eyes. Maximum vitreoretinal adhesion in adult human eyes (30–79 years old) was greater in the equator than in the posterior pole, especially at young ages (30–39 years old). After 60 years of age, there was a significant drop in equatorial adhesion that may be protective to the retina by facilitating vitreous detachment during liquefaction. In immature (premature, neonatal, and young lamb) and mature (young adult) sheep eyes, there was no significant difference in posterior vitreoretinal adhesion, but maximum equatorial adhesion in mature eyes was 2 times greater than immature eyes. This may be caused by the extension of the vitreous base during development. These data are the first quantitative measurements of vitreoretinal adhesion, and will be useful in the development of computational models for simulating posterior vitreous detachment or ocular trauma. The methods and technology developed for this study can be used to evaluate mechanisms of adhesion, and assess the efficacy of enzymes to remove or reduce vitreoretinal adhesion for surgical interventions.

## Data availability

All data used in this manuscript is available via doi: 10.7278/S5BK19H3 located within the University of Utah Research Data Repository (https://hive.utah.edu).

## Author contributions

CC performed testing, sectioning, preliminary analysis and manuscript writing. JC assisted with all aspects of the data analysis and manuscript writing. BC was responsible for the study conception and design, data interpretation, and manuscript editing.

### Conflict of interest statement

The authors declare that the research was conducted in the absence of any commercial or financial relationships that could be construed as a potential conflict of interest.
